# Traumatic fibroma

**DOI:** 10.11604/pamj.2015.21.220.7498

**Published:** 2015-07-27

**Authors:** Prashanth Panta

**Affiliations:** 1 Department of Oral Medicine and Radiology, MNR Dental College and Hospital Narsapurroad, Sangareddy, Telangana, India

**Keywords:** Fibroma, trauma, connective tissue

## Image in medicine

A 29 year old female presented with a growth in relation to the right cheek mucosa (A). On inquiry, she revealed that it had been gradually increasing in size, since she first noticed 4 months earlier. History revealed that she habitually bit her cheek. Constitutional symptoms and lymph node enlargement were absent. The nodule was 2 X 2 cm in size, yellowish in color, located on the right buccal mucosa, along the line of occlusion corresponding to the sharp cusps of maxillary 1^st^ and 2^nd^ molar (B). On palpation, it was nontender, firm and pedunculated. The differential diagnosis included fibroma, myxoma, mucocele, lipoma and pleomorphic adenoma. An excisional biopsy was performed and the microscopic features suggested “Fibroma”. The patient was recalled after 2 months to evaluate healing, which was uneventful (C). Fibromas are benign tumors of connective tissue origin. In the oral cavity, they occur in response to irritation from local trauma, and the most common sites are buccal mucosa and tongue. Their size usually ranges from 0.5 cm to 1.5 cm. They are asymptomatic, dome shaped, smooth surfaced, sessile or pedunculated masses that usually occur in females between 20-50 years. The recurrence rate is very low, but when it occurs, it may be due to the persistence of offending irritant.

**Figure 1:**
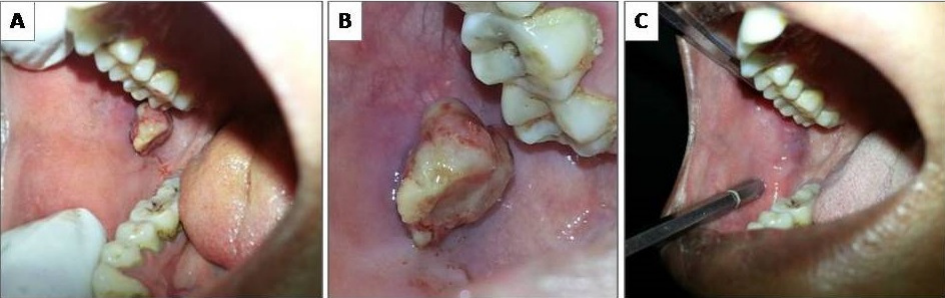
An intraoral traumatic fibroma on the right buccal mucosa (A, B, C)

